# Editorial: Environmental toxicity in reproduction

**DOI:** 10.3389/fphys.2023.1351594

**Published:** 2023-12-19

**Authors:** Jiahui Ding, Yong Song, Chunyan Liu, Marianna Sadagurski

**Affiliations:** ^1^ CS Mott Center for Human Growth and Development, Department of Obstetrics and Gynecology, Wayne State University, Detroit, MI, United States; ^2^ Department of Obstetrics, Gynecology and Reproductive Biology, Michigan State University, Grand Rapids, MI, United States; ^3^ Tongji Medical College, Institute of Reproductive Health, Huazhong University of Science and Technology, Wuhan, China; ^4^ Integrative Biosciences Center (IBio), Department of Biological Sciences, Wayne State University, Detroit, MI, United States

**Keywords:** toxicity, pregnancy, reproduction, fertility, environment

To achieve a successful pregnancy, the critical events such as ovulation, implantation and placentation must be tightly regulated. Pregnancy represents a unique physiological state characterized by the continuous adaption of the maternal immune system in response to the dynamic stages of fetal development and signals originating from both internal and external environments. Exposure to environmental toxicants, including heavy metals, air pollutants, and industrial chemicals, during this delicate period can lead to adverse outcomes, including miscarriage, preterm birth, preeclampsia, low birth weight, and long-term health issues such as neurological disorders in offspring. Exposure to environmental toxicants not only affects pregnancy but also compromises other reproductive functions, such as oogenesis, leading to potential complications in subsequent pregnancy. It is now widely acknowledged that increasing exposure to environmental toxicants both prior to and during pregnancy poses a substantial threat to the initiation and sustenance of a healthy pregnancy as well as the subsequent wellbeing of the offspring. However, the actions and underlying mechanisms of these environmental toxicants are still poorly understood. To contribute to greater understanding of this aspect, in this Research Topic, we have assembled a unique collection of interrelated studies which shed light on the significant effects of various toxicants on female reproduction, covering fertility and pregnancy from both experimental and conceptual perspectives.

In the first article, Geng et al. focus on microplastics (MPs) and nanoplastics (NPs), which are emerging environmental pollutants known for their reproductive toxicity in females. Regarding the toxic mechanism of MPs and NPs, it is revealed that MPs/NPs may act as endocrine disruptors, impacting hypothalamic pituitary gonadal axis, hormone synthesis and release. Additionally, the reproductive toxicity of MPs/NPs encompasses perturbations in bioenergy utilization, the generation of reactive oxygen species (ROS), DNA damage and genetic modifications. Importantly, MPs/NPs have the potential to translocate to oocytes, placenta, and fetus, disrupting the delicate immune balance at the maternal-fetal interface while also altering the fetal immune system development. Neurological dysfunction, metabolic disorders, developmental abnormalities, and subfertility in offspring haven been indicated in the animal models. However, it is worth noting that in the natural environment, MPs/NPs seldom play a solitary function. They are suggested to serve as carries for other environmental contaminants, such as endocrine disrupting chemicals and heavy metals. An additional challenge in replicating natural exposure situations is the dosage, as the concentration of microplastics in the environment can be as low as less than 1 μg/L, which is significantly below the concentrations employed in experimental settings. Overall, this comprehensive review paper summarizes the outcomes of exposure to MPs/NPs, along with their potential mechanisms. It also highlights the limitations of current studies and outlines future directions for the related research, offering valuable insights into the key questions the researchers still need to address.

In 2022, the World Health Organization reported a global infertility prevalence of approximately 17.5%, with ovulatory disorders accounting for around 25% of all infertility diagnoses. Oogenesis, a pivotal process in female ovulatory function, significantly influences fertility, and abnormalities at any stage can result in defective oogenesis. Increasing evidence suggests that exposure to environmental toxicants, both through occupational exposure and ubiquitous chemicals, has detrimental effects on oocyte development. However, the precise impact of environmental toxicants on oogenesis remains largely unknown. Yao et al. systematically classified environmental toxicants, including heavy metals, cigarette smoke, and agricultural and industrial pollutants, offering a structured framework to understand their varied effects on oogenesis. This categorization, together with an exploration of the underlying genetic and molecular mechanisms, provides a subtle understanding of how these toxins disrupt the complex processes governing oogenesis. For instance, Zinc oxide nanoparticles (ZnO NPs), widely used in industrial and commercial products, inhibit oocyte meiosis and reduce oocyte quantity and quality. The involved mechanisms include mitochondrial and endoplasmic reticulum stress, leading to oocyte apoptosis and autophagy, disruption of the cytoskeletal structure, and increased levels of reactive oxygen species. Importantly, this review emphasizes the growing concern surrounding environmental toxicants exposure and its identified role as a significant risk factor for oogenesis in women. It also highlights calls for further research to fully explore in detail mechanisms and identify biomarkers capable of assessing exposure levels and predicting reproductive outcomes.

In the third article, Su et al. focus on reproductive toxicity of cadmium (Cd) exposure by using a new fish model, Gobiocypris rarus. Cd is a heavy metal found in the natural environment. Exposure to high concentrations of cadmium can severely affect fish reproduction. However, the effect of cadmium at concentrations related to the water environment on the reproductive function of parental fish is unclear. The rare minnow, Gobiocypris rarus, a native Chinese fish species, is a new fish model for monitoring the water environment in China. Su et al. exposed male and female fathead minnows to cadmium at 0, 5 and 10 μg/L for 28 days and then paired them to spawn. They observed that Cd exposure at 5 or 10 μg/L impaired gonadal development and reproductive capacity in rare minnow. Although the limit of Cd concentration in water is 5 μg/L according to Chinese fishery water quality standards (GB 11607-89), Cd exposure at 5 μg/L is still detrimental to the reproductive activity of fish. Thus, this study has positive implications for further controlling the limited cadmium concentrations in water to protect the reproductive health of not only fish but also humans.

In summary, by covering a broad range of Research Topic at experimental and conceptual levels, this Research Topic highlights the multiscale and multifaceted complexities of environmental toxicity in fertility and pregnancy. It is foreseeable that ongoing investigations in this field will contribute to a better understanding of these complexities, ultimately leading to the development of innovative preventive and therapeutic approaches for enhancing the wellbeing of pregnant women and their offspring.

